# Preparation and Characterization of Antibacterial Polyvinyl Alcohol Films Containing *Syzygium aromaticum* Essential Oil

**DOI:** 10.3390/polym18060714

**Published:** 2026-03-15

**Authors:** Arzu Özgen

**Affiliations:** Department of Medical Services and Techniques, Vocational School of Health Services, Istanbul Gelisim University, Istanbul 34310, Türkiye; aozgen@gelisim.edu.tr

**Keywords:** PVA, *Syzygium aromaticum*, antibacterial activity, antibacterial polymer, molecular docking

## Abstract

The resistance of pathogenic bacteria to antimicrobial agents is currently one of the most significant health challenges. Polymers and nano-polymer composites with antimicrobial properties are widely used, particularly in hospitals, biocompatible implants, and the medical device industry. *Syzygium aromaticum* (clove) contains several bioactive compounds, including potent antioxidants and antimicrobials, which confer antioxidant, antibacterial, and antiseptic properties. For this purpose, polyvinyl alcohol (PVA) films were produced at three different concentrations using a direct integration method and doped with clove essential oil. The spectral, structural, and thermal properties of the produced films were analyzed, and their antibacterial activity against *Klebsiella pneumoniae* was tested. Fourier Transform Infrared Spectroscopy (FTIR) results confirm that the structural integrity of the PVA matrix is preserved and that the essential oil is physically trapped within the polymer network. Overall, the Differential Scanning Calorimetry (DSC) results confirm that *Syzygium aromaticum* essential oil (SAEO) acts as an effective plasticizer in PVA films, significantly modifying the glass transition behavior and enhancing polymer chain mobility in a concentration-dependent manner. The Dynamic Mechanical Analysis (DMA) results, supported by DSC analysis, clearly demonstrate that SAEO acts as an effective plasticizing agent in PVA films by increasing molecular mobility, lowering the glass transition temperature (Tg), and promoting thermally induced deformation. The concentration-dependent increase in the diameter of the inhibition zone of essential-oil-added films showed that their antibacterial efficacy increased as the *S. aromaticum* essential oil content increased (0.5%, 0.75%, and 1.0%). Additionally, molecular docking was performed to examine interactions between selected virulence proteins of *K. pneumoniae* and the main components of clove essential oil. As a result, *S. aromaticum* essential oil conferred antibacterial properties to the polyvinyl alcohol films without significantly altering their transparency and thermal properties.

## 1. Introduction

Microbes can develop defense strategies, known as resistance mechanisms, to survive antibiotics and antifungal drugs [1]. Antimicrobial resistance (AMR) arises through evolutionary processes by which microorganisms, including bacteria, fungi, parasites, and viruses, acquire resistance to antimicrobial drugs such as antibiotics, which are commonly used for treatment [2]. The misuse or overuse of antimicrobial agents in healthcare, veterinary, and agricultural settings contributes to this resistance [3]. As a result, treating human and animal diseases becomes more challenging. Increased resistance heightens the risk of disease spread, prolongs illness duration, extends hospital stays, necessitates more expensive treatments, and raises mortality rates [4].

Polyvinyl alcohol (PVA) is a water-soluble material with excellent chemical resistance and physical properties [5]. Furthermore, its non-toxic effects, high film-forming capacity, chemical resistance, and biodegradable/biocompatible nature give it potential for widespread use in commercial medical applications [6]. PVA is used in the synthesis of contact lenses, dressings, medicines (for drug delivery), corneal implants, heart valves, and cartilage tissue implants [7].

Natural antimicrobial agents derived from plants are primarily obtained from substances such as plant extracts, including essential oils [8]. The addition of essential oils to PVA-based films to create antimicrobial materials is a topic that is widely researched today [9,10,11,12].

*S. aromaticum*, commonly known as clove (also referred to as *Caryophyllus aromaticus*, *Caryophyllus silvestris*, *Eugenia caryophyllus*, *Jambosa caryophyllus*, and *Myrtus caryophyllus*), is a member of the Myrtaceae family. It originates from Indonesia and is recognized as one of the most valuable spices, ranking second in importance in world trade [13]. Clove essential oil contains a variety of bioactive compounds, which include potent antioxidants and antimicrobials. These components impart several beneficial properties to clove oil: it acts as an antioxidant, antibacterial, antiseptic, pesticidal, analgesic, and anticarcinogenic agent. Furthermore, the U.S. Food and Drug Administration (FDA) classifies clove oil as generally recognized as safe (GRAS) for use as a food additive [14]. Due to these properties, clove oil is valuable across multiple sectors, including food, biomedical applications, packaging, hygiene products, cosmetics, and pharmaceuticals [15].

*Klebsiella pneumoniae* (*K. pneumoniae*) is a Gram-negative bacterium and an opportunistic pathogen that can cause a wide range of infections in humans, including urinary tract infections, pneumonia, sepsis, and infections of wounds and soft tissue. Another infectious aspect of *K. pneumoniae* is the biofilm it can form on medical devices (catheters and endotracheal tubes), posing a significant source of infection in patients with these devices [16,17]. The World Health Organization (WHO) included *K. pneumoniae* on its 2024 Bacterial Priority Pathogens List, highlighting the urgent need to develop new antibiotics to treat infections caused by this bacterium [18]. Virulence factors are crucial components that allow bacteria to cause infections, evade host defenses, and lead to disease. Like other pathogenic microorganisms, “*K. pneumoniae*” employs a variety of virulence factors to enhance its survival and promote disease development, including the capsule, lipopolysaccharide (LPS), siderophores, type I and III fimbriae, outer membrane proteins, and type VI secretion system [19]. The virulence factor of *K. pneumoniae*, coded Protein Data Bank (PDB) ID 2ZYG, is expressed by the *gnd* gene. This gene encodes gluconate-6-phosphate dehydrogenase and can enhance the production of capsular polysaccharides, which are crucial for the virulence of *K. pneumoniae* during infection [20,21]. The capsule is a polysaccharide matrix that surrounds the cell and consists of strain-specific capsular polysaccharides known as K antigens. The *gnd* gene, which is also among the genes involved in capsule production, is located on the chromosomal cps operon [22]. It encodes NADP-dependent 6-phosphogluconate dehydrogenase, which participates in the pentose phosphate pathway. This enzyme converts 6-phosphogluconate to ribulose-5-phosphate and generates NADPH [20,23]. The virulence factors encoded by PDB IDs 9AT9 and 3U4K are encoded by the *fimH* and *mrkD* genes, respectively. These genes, which encode type 1 and type 3 fimbrial adhesion proteins, respectively, are responsible for binding to host cells and are the main fimbriae involved in biofilm formation [24], adhesion, and tissue affinity in *K. pneumoniae* [25]. The *fimH* gene (type 1 fimbria) has been reported to be present in 86.5% of isolates, and the *mrkD* gene (type 3 adhesion protein) in 88.5% of isolates [24]. Type 1 fimbriae enable adhesion to many epithelial cell types, especially the bladder epithelium [26].

Hygiene is important for medical devices that improve quality of life, such as implants, prostheses, catheters, contact lenses, and pacemakers [7], which are susceptible to microbial contamination [27]. Therefore, the synthesis and development of new materials to prevent infections caused by antibiotic-resistant microorganisms is becoming increasingly important. The increasing global prevalence of multidrug-resistant (MDR) Gram-negative bacteria is significantly increasing the burden of chronic and treatment-resistant infections, largely due to biofilm formation. Biofilm structures confer significant resistance to antimicrobial agents and immune response mechanisms, presenting a major clinical challenge [28]. This necessitates the development of non-antibiotic approaches to infection control. Surface coating or matrix systems capable of suppressing biofilm formation are needed to prevent medical device-associated infections. In this context, PVA-based alternative products with biofilm-anti-responsible properties offer a noteworthy area of research from a translational perspective [7,16,29].

The main objective of this study is to develop and evaluate the potential of a biocompatible, antibiotic-alternative, and antibacterial film candidate suitable for producing medical equipment such as catheters, endotracheal tubes, and wound dressings. This is achieved by incorporating *S. aromaticum* essential oil (SAEO) as a filler into a PVA polymer matrix. The novelty of this study lies in incorporating SAEO into a PVA matrix, preserving the polymer’s fundamental structural properties, and evaluating the resulting films for their physicochemical characteristics and antibacterial activity. To evaluate the effectiveness of the produced films, their spectral, structural, and thermal properties were analyzed, and their antibacterial activity against *K. pneumoniae* was tested. Additionally, molecular docking was performed to examine interactions between selected virulence proteins of *K. pneumoniae* and the main components of SAEO.

## 2. Experimental Section

### 2.1. Materials

Polyvinyl alcohol (PVA; molecular weight 89,000–98,000 g/mol; degree of hydrolysis ≥ 99%) and glutaraldehyde (25% aqueous solution) were purchased from Merck (Darmstadt, Germany). *Syzygium aromaticum* essential oil (SAEO) was purchased from a local producer (Bade Natural, İstanbul, Türkiye). *Klebsiella pneumoniae* ATCC 700603, used in antibacterial activity experiments, was kept in our laboratory.

### 2.2. Synthesis and Preparation Studies

#### Preparation of Antibacterial Films

**Figure 1:**
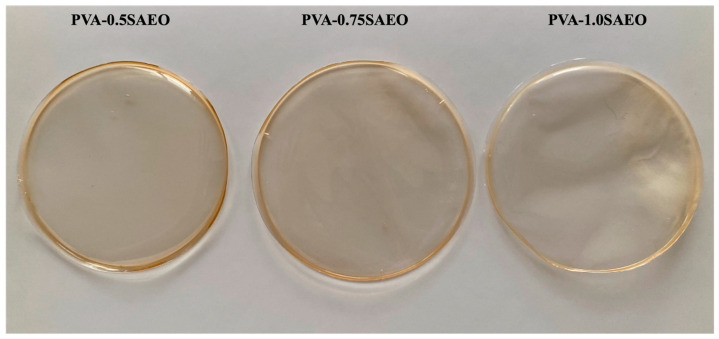
The images of the polymer (PVA) films doped with 0.5%, 0.75%, and 1% SAEO.

PVA polymer is suitable for use as a surfactant in film production processes due to its combination of hydrophilic and hydrophobic functional groups, and thus was used as a matrix in films prepared via a two-step direct incorporation method. In the first step, 4 g of PVA was added to 46 mL of deionized water (dH_2_O) and stirred for four hours at 95 °C using a WiseStir MSH-20D hotplate stirrer (Daihan Scientific Co., Ltd., Wonju, Korea) set to 550 rpm until the PVA dissolved completely. During this stage, 50 µL of glutaraldehyde was added to the solution to promote cross-linking, and the mixture was stirred for an additional hour [30]. Glutaraldehyde was employed as a bifunctional crosslinking agent capable of reacting with the hydroxyl groups of PVA, forming acetal linkages under thermal conditions. This crosslinking step was introduced to enhance the mechanical stability, water resistance, and dimensional integrity of the films, particularly considering the plasticizing effect of SAEO. The presence of crosslinks helps to maintain structural cohesion within the polymer network while allowing for controlled flexibility [31,32]. In the second step, various concentrations (250, 375, and 500 µL) of SAEO (pure) were added to the cooled PVA solution and mixed for one hour. Finally, 15 mL of the homogeneous mixture was poured into Petri dishes (9 cm) at room temperature and left to dry in an oven for 48 h [11]. The images of the polymer films doped with 0.5%, 0.75%, and 1.0% SAEO that were obtained through the direct incorporation method are shown in Figure 1.

### 2.3. Analysis and Testing Studies

#### 2.3.1. Structural Characterization of the Addition of SAEO to PVA

The chemical structures of neat PVA and PVA film samples containing different concentrations of SAEO (0.50, 0.75, and 1.00 wt%) were analyzed using Fourier transform infrared spectroscopy (FTIR) to investigate possible changes in functional groups and molecular interactions. FTIR measurements were carried out using a spectrometer (PerkinElmer Inc., Waltham, MA, USA) equipped with an ATR (Attenuated Total Reflection), and the spectra were recorded in the wavenumber range of 4000–400 cm^−1^ in percent transmittance (%T) mode with a spectral resolution of 4.0 cm^−1^. The film samples were analyzed directly in their solid film form without any chemical pretreatment, and the obtained FTIR spectra were compared to elucidate the interactions between neat PVA and PVA films incorporating SAEO, as well as to assess the influence of the essential oil on the polymer matrix [33].

#### 2.3.2. Thermal Analysis of Neat PVA and Addition of SAEO to PVA

The thermal properties of neat PVA and PVA film samples containing different concentrations of SAEO (0.5, 0.75, and 1.0 wt%) were investigated using differential scanning calorimetry (DSC) (SII Nanotechnology, Hitachi ExStar 6200, High-Tech Science Corporation, Tokyo, Japan) equipped with a Thermo Scientific Intracooler EK90C/SII (Thermo Fisher Scientific Inc., Waltham, MA, USA) electrical cooling device. DSC analyses were performed using aluminum pans with approximately 5–7 mg of each sample, and an empty aluminum pan served as the reference [33].

The measurements were carried out over a temperature range of −40 to 140 °C at a constant heating rate of 10 °C/min to ensure comparable evaluation of the thermal transitions of the samples. The temperature program was applied in ramp mode, and a 2 min isothermal holding period at the initial temperature was introduced to allow for thermal equilibration prior to heating. Throughout the analysis, nitrogen (N_2_) was employed to provide an inert atmosphere and prevent oxidative degradation, while the oxygen flow was kept off.

During the DSC measurements, data were recorded as heat flow (µW). The resulting DSC thermograms were used to evaluate the glass transition temperature (Tg), melting behavior, and other relevant thermal events of neat and SAEO-incorporated PVA films, enabling a comparative assessment of the effect of essential oil content on the thermal characteristics of PVA.

#### 2.3.3. Creep Behaviors of PVA and SAEO-Loaded Films by DMA

Solid-state viscoelastic properties of film samples were characterized by creep tests performed with a dynamic mechanical analyzer (DMA, SII Nanotechnology, ExStar DMS 6100, Hitachi High-Tech Science Corporation, Tokyo, Japan) at 25 °C, where an instantaneous stress of 3 MPa was loaded in uniaxial tension mode and time-dependent strain was monitored for 30 min. Then, the creep strain data were converted to stress–strain (SS) data with DMA software (MuseJobe, Standard Analysis) and the elasticity (elastic modulus values) was calculated using the tangential slope of the SS plots [33].

#### 2.3.4. The Optical Properties of Addition of SAEO to PVA

The optical properties of neat PVA and Polyvinyl alcohol-*Syzygium aromaticum* essential oil (PVA-SAEO) films were evaluated using UV–visible spectroscopy (ColorFlex EZ, HunterLab, Reston, VA, USA), and the film samples were cut into rectangular pieces and placed directly in the sample holder of the spectrophotometer. The UV–Vis transmittance spectra were recorded over the wavelength range of 200–900 nm at room temperature, with air used as the reference background [34].

The optical behavior of the films was expressed as percentage transmittance (%T) as a function of wavelength. All measurements were performed at least in triplicate to ensure reproducibility, and representative spectra were reported.

#### 2.3.5. Antimicrobial Activity Test of Antibacterial Films

The well-diffusion method was used to determine the antibacterial activities of the prepared PVA-SAEO film samples on *K. pneumoniae* ATCC 700603. Neat PVA and SAEO were used as the negative control [11], and FOX: cefoxitin (30 µg/mL) (Bioanalyse, Ankara, Türkiye) was used as the positive control for *K. pneumoniae* ATCC 700603, where the *K. pneumoniae* was grown in 16 h Müller–Hinton broth (MHB) (Biolife, Italiana S.r.l., Milan, Italy). The bacterial strain was cultured for 16 h at 37 °C on Müller–Hinton agar (MHA) (Biolab Diagnostics Ltd., Budapest, Hungary), and the densities of the bacterial suspensions were adjusted to the 0.5 McFarland standard (1.5 × 10^8^ CFU/mL) by diluting with MHB at a 1:100 ratio, as per Clinical and Laboratory Standards Institute (CLSI) guidelines, 2012 [35]. Subsequently, 100 µL of the bacterial suspension was uniformly spread onto MHA Petri dishes using a sterile spreader. Then, using a sterile pipette tip, a hole with a diameter of 6 mm was punched aseptically. Circular pieces with a diameter of 6 mm were prepared from neat PVA and PVA-SAEO films prepared at different concentrations (0.5%, 0.75%, and 1%) [11,36,37]. After incubating the plates at 37 °C for 24 h, the diameters of the inhibition zones observed in the Petri dishes were measured in millimeters. Each experimental trial was repeated three times.

### 2.4. In Silico Studies

#### 2.4.1. A Bioinformatics Study on Various Virulence Factors of *K. pneumoniae*

Amino acid sequences of the proteins belonging to *K. pneumoniae* virulence factors that they encode were obtained from the Protein Database of NCBI (https://www.ncbi.nlm.nih.gov/protein/) (accessed on 23 December 2025). Information on the conserved domains of virulence proteins was obtained from the Conserved Domain Search Service (CD-Search) NCBI (https://www.ncbi.nlm.nih.gov/Structure/cdd/wrpsb.cgi) (accessed on 24 December 2025). The predicted subcellular location of the virulence gene was displayed using the online server DeepLocPro-1.0 (https://services.healthtech.dtu.dk/services/DeepLocPro-1.0/) (Accessed on 24 December 2025).

#### 2.4.2. Molecular Docking Methodology

**Figure 2:**
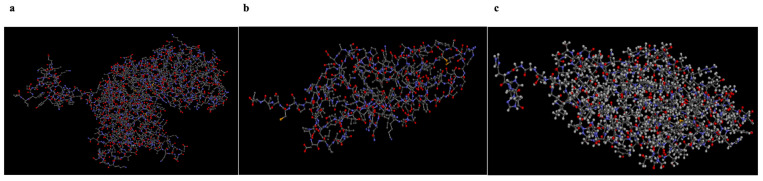
Three-dimensional molecular structures of 2ZYG (**a**), 3U4K (**b**), and 9AT9 (**c**) macromolecules. The colors symbolizing the atoms are given in Table 1.

The protein structures, identified by PDB IDs 2ZYG, 3U4K, and 9AT9, were obtained from the RCSB PDB. Each protein molecule was optimized using AutoDock’s Protein Preparation Wizard (PyRx-Virtual Screening Tool, PyRx-0.8). The PyRx has built-in AutoDock, AutoDock Wizard, Vina Wizard, and Open Babel. Water molecules were removed from the protein structures used for molecular docking. Figure 2 shows the 3D structures of proteins in a ball-and-stick style.

**Figure 3:**
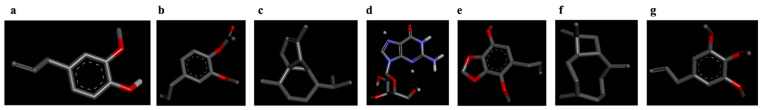
Three-dimensional molecular structures of eugenol (**a**), eugenyl acetate (**b**), α-cubebene (**c**), guanosine (**d**), apioline (**e**), β-caryophyllene (**f**), and elemicin (**g**) molecules. The colors symbolizing the atoms are given in Table 1.

Ligand structures of eugenol, eugenyl acetate, α-cubebene, guanosine, apioline, β-caryophyllene, and elemicin, identified as PubChem CIDs 3314, 7136, 442359, 135398635, 10659, 5281515, and 10248, respectively, were obtained from the National Library of Medicine. They were then converted to PDBQT files using the PyRx virtual screening tool (PyRx-Virtual Screening Tool, PyRx-0.8) to generate the atomic coordinates and optimize ligands. Figure 3 shows the 3D structures of the ligands prepared for molecular docking.

#### 2.4.3. Docking Protocol Used in Autodock Vina

In this study, molecular docking was performed using the free version of PyRx, and each docking was carried out according to standard AutoDock procedures [38]. The AutoGrid engine in Pyrex was used to generate the grid configuration file for the grid parameters and to predict amino acids that interact with the ligands in the protein’s active site. The ligand with the most significant binding affinity was thought to have the most considerable binding energy, which was also the most negative. Finally, analyses and images were obtained using the BIOVIA Discovery Studio 2024 Client program. The colors symbolizing the atoms shown in Figure 2 and Figure 3 are given in Table 1.

**Table 1:** Colors symbolizing atoms.

Element	Hydrogen	Carbon	Oxygen	Sulfur	Nitrogen
Color	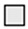	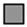	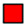	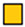	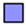

## 3. Results and Discussion

### 3.1. Structural Characterization of Neat PVA and Addition of SAEO to PVA

**Figure 4:**
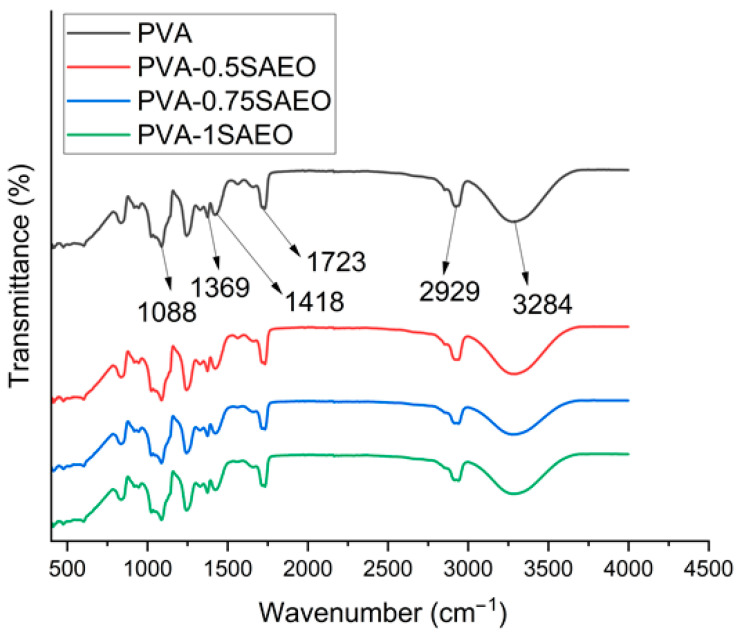
FTIR spectra of neat PVA, PVA-0.5SAEO (0.5%), PVA-0.75SAEO (0.75%), and PVA-1SAEO (1.0%) films.

The FTIR spectra of neat PVA and PVA film samples containing different concentrations of SAEO were comparatively evaluated to investigate the possible interactions between the polymer matrix and the essential oil components (Figure 4).

In the spectrum of the neat PVA film, the broad absorption band observed in the range of 3200–3600 cm^−1^ is attributed to the strong hydrogen bonding of hydroxyl (–OH) groups along the PVA chains. This band is a characteristic indicator of the hydrophilic nature of PVA and its extensive intermolecular interactions. The absorption bands appearing around 2940–2910 cm^−1^ correspond to the stretching vibrations of aliphatic –CH and –CH_2_ groups. A weak band detected at approximately 1730–1710 cm^−1^ is associated with the C=O stretching vibrations of residual acetate groups present in partially hydrolyzed PVA. The bands in the 1420–1320 cm^−1^ region are assigned to –CH_2_ bending vibrations, while those observed at around 1140–1080 cm^−1^ correspond to C–O–C and C–O stretching vibrations. These peaks are well defined and widely reported as characteristic bands of PVA in the literature [30,39,40].

Upon incorporation of *S. aromaticum* essential oil into the PVA matrix, the FTIR spectra did not exhibit pronounced wavenumber shifts; however, slight band broadening within the 3200–3600 cm^−1^ region was observed. This region corresponds to the O–H stretching vibrations of PVA, which are highly sensitive to hydrogen bonding interactions. Although the spectral changes are subtle, the observed broadening is consistent with possible intermolecular interactions between the phenolic hydroxyl groups of eugenol—the major component of SAEO—and the hydroxyl groups of polyvinyl alcohol chains. Similar behavior has been reported in essential-oil-incorporated polymer systems, where hydrogen bonding results in modest spectral modifications rather than distinct peak shifts [41].

In the 1600–1510 cm^−1^ region, weak contributions overlapping with the characteristic PVA bands were detected. This region is typically associated with aromatic C=C stretching vibrations of eugenol [42]. However, due to the dominant spectral features of the PVA substrate, these bands cannot be unambiguously assigned without spectral subtraction or further deconvolution analysis. Therefore, the presence of essential oil within the polymer matrix is interpreted in a qualitative manner, supported by the appearance of minor intensity variations and literature-reported spectral signatures of eugenol-containing systems. Similarly, variations in the 1260–1030 cm^−1^ region may arise from overlapping C–O stretching vibrations of both PVA and essential oil components. Given the spectral overlap inherent to polymer–essential oil composites, substrate interference is expected, and the FTIR findings should be interpreted as supportive rather than definitive evidence of intermolecular interactions.

Overall, the FTIR results demonstrate that SAEO interacts with the PVA matrix predominantly through hydrogen bonding and physical interactions rather than through the formation of new chemical bonds. The absence of new functional group peaks indicates that the structural integrity of the PVA matrix is preserved and confirms that the essential oil is physically entrapped within the polymer network.

### 3.2. Thermal Analysis of Neat PVA and Addition of SAEO to PVA

**Figure 5:**
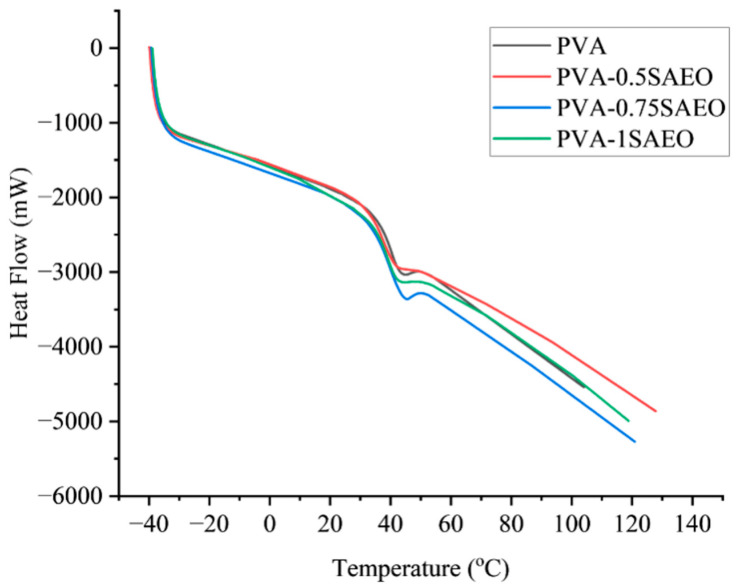
DSC thermograms of neat PVA and PVA-0.5SAEO, PVA-0.75SAEO, and PVA-1SAEO films.

The thermal behavior of neat PVA and PVA films containing SAEO (0.5, 0.75, and 1 wt%) was evaluated by DSC, and the corresponding thermograms are shown in Figure 5. All samples exhibit a broad endothermic transition in the temperature range of ~40–55 °C, which is characteristic of a glass transition-related thermal relaxation process in PVA-based systems [43].

For neat PVA, the endothermic event centered at approximately 45–50 °C is attributed to the onset of segmental mobility in the amorphous regions, associated with the partial disruption of intermolecular hydrogen bonding between PVA chains. Although the glass transition temperature (Tg) of fully hydrolyzed PVA is commonly reported at ~80–90 °C, it is well established that the presence of absorbed or bound water significantly plasticizes the polymer, leading to a pronounced reduction in the apparent Tg measured by DSC. Accordingly, Tg values in the range of 35–60 °C have frequently been reported for PVA films prepared under ambient conditions [44,45].

The incorporation of SAEO markedly alters the thermal response of PVA films. Compared with neat PVA, essential-oil-loaded samples display a more pronounced and broadened glass transition region, indicating enhanced polymer chain mobility. This effect becomes increasingly evident with rising essential oil content, particularly for the 0.75 wt% and 1 wt% formulations, suggesting a concentration-dependent plasticizing action of the additive [46].

The observed reduction and broadening of the glass transition region can be attributed to the molecular interactions between PVA and SAEO. Eugenol, SAEO’s major constituent, is a phenolic compound containing hydroxyl groups capable of forming hydrogen bonds with PVA chains. These interactions weaken PVA–PVA hydrogen bonding, increase free volume, and facilitate segmental motion at lower temperatures. Similar Tg depression phenomena have been extensively reported for PVA systems containing low-molecular-weight plasticizers or essential oils [47,48].

Among the investigated compositions, the film containing 0.75 wt% SAEO exhibits the most distinct and well-defined glass transition behavior, implying an optimal balance between polymer–additive interactions and matrix homogeneity. In contrast, the further broadening observed at 1 wt% essential oil loading may indicate the onset of microstructural heterogeneity or partial phase separation, as previously reported for PVA matrices incorporating higher amounts of hydrophobic additives [12,49].

No melting endotherm was detected for any sample within the investigated temperature range (up to 140 °C), consistent with the known melting temperature of PVA (typically 180–230 °C). Therefore, the thermal transitions observed in this study are attributed exclusively to amorphous phase relaxation processes rather than crystalline melting [45].

It should be noted that the essential oil concentrations reported correspond to the nominal amounts added during film preparation. Due to the volatile nature of essential oils and the possibility of partial surface migration during solvent casting, the actual retained amount within the polymer matrix may slightly differ from the nominal loading. Consequently, the thermal response may not exhibit a strictly linear correlation with the incorporated oil percentage. Nevertheless, the systematic Tg depression and broadening observed with increasing SAEO content indicate effective interaction and functional incorporation of the oil within the PVA matrix.

Overall, the DSC results confirm that SAEO acts as an effective plasticizer in PVA films, significantly modifying the glass transition behavior and enhancing polymer chain mobility in a concentration-dependent manner.

### 3.3. Creep Behaviors of PVA and SAEO-Loaded Films by DMA

**Figure 6:**
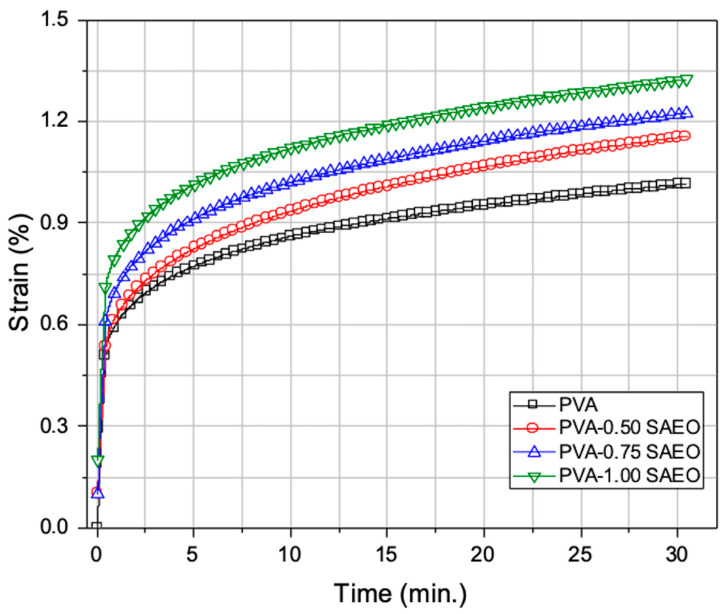
The time-dependent strain (%) behaviors of neat PVA and PVA-0.5SAEO, PVA-0.75SAEO, and PVA-1SAEO films.

Figure 6 shows the creep curves, i.e., time-dependent strain (%) behaviors, of neat PVA and *S. aromaticum* essential oil (SAEO)-incorporated PVA films.

It was found that the neat PVA reached the lowest final strain value within 30 min, at approximately 1.0%, which can be attributed to its strong intermolecular hydrogen bonding and semi-crystalline structure, providing high resistance to creep deformation [50,51].

Upon incorporation of SAEO, a noticeable increase in strain is observed in the film samples, which becomes progressively more pronounced as the SAEO content increases from 0.5 to 1.0 wt%. This behavior is characteristic of a plasticizing effect, where low-molecular-weight additives reduce intermolecular forces and increase the free volume within the polymer matrix, leading to enhanced chain mobility [52,53,54]. However, the magnitude of this increase remains within a moderate range, suggesting that the overall structural integrity of the PVA matrix is largely preserved.

The observed plasticization effect, rather than presenting a drastic alteration in the polymer network, is in good agreement with the DSC results (Figure 5), which also show a slight decrease in Tg upon SAEO addition. Although minor deviations from strict proportionality between strain magnitude and nominal oil content may arise from the local distribution heterogeneity of the essential oil within the matrix, the overall trend clearly reflects a concentration-dependent plasticization behavior. Such consistency between DMA and DSC data has been widely reported for plasticized PVA systems and confirms the reliability of the observed thermal transitions [51,55].

At higher temperatures, SAEO-incorporated films, particularly those containing 0.75 and 1.0 wt% SAEO, exhibit a steeper increase in strain, reflecting reduced dimensional stability and increased viscoelastic flow. Nevertheless, this effect might become significant primarily at elevated temperatures and does not indicate severe thermal degradation or structural collapse. This behavior is attributed to the combined effects of plasticization and a slight reduction in intermolecular cohesion within the semi-crystalline structure of PVA, which has been previously observed in PVA films containing essential oils or other bio-based plasticizers [53,56,57]. The increased deformability at elevated temperatures indicates that SAEO effectively enhances the flexibility of the PVA matrix while maintaining acceptable thermo-mechanical stability within the practical application range.

Overall, the DMA results, supported by DSC analysis, clearly demonstrate that SAEO acts as an effective plasticizing agent in PVA films by increasing molecular mobility, lowering Tg, and promoting thermally induced deformation. Similar correlations between strain behavior, Tg reduction, and plasticizer concentration have been extensively reported in the literature for PVA-based composite and bioactive films [52,54,57].

The elastic modulus values of samples calculated with the creep-converted stress–strain curves are listed in Table 2, which shows that the PVA exhibits the highest modulus value as expected because it shows the lowest creep strain. SAEO incorporation decreased the elastic modulus value of PVA by 13–15%, which is consistent with the previously mentioned conclusion on the plasticizing effect of SAEO.

### 3.4. The Optical Properties of Addition of SAEO to PVA

**Figure 7:**
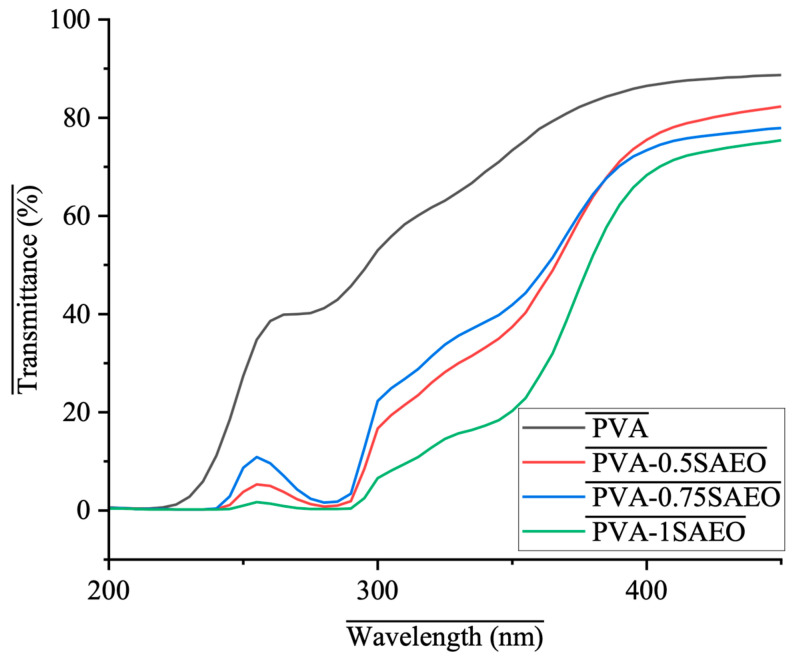
UV–Vis transmittance spectra of neat PVA and PVA-0.5SAEO, PVA-0.75SAEO, and PVA-1.0SAEO films.

The UV–Vis transmittance spectra of neat PVA and PVA films incorporated with different concentrations of SAEO (0.5, 0.75, and 1.0 wt%) are presented in Figure 7, providing valuable insight into the optical transparency and UV barrier performance of the developed films.

Neat PVA film exhibited the highest transmittance values within the measured spectral range shown in Figure 7, confirming the inherently high optical transparency of polyvinyl alcohol, which is attributed to its homogeneous polymeric structure and absence of strong chromophoric groups capable of absorbing UV radiation. In contrast, the incorporation of SAEO resulted in a noticeable decrease in transmittance in the ultraviolet region of the recorded spectrum, with the effect becoming progressively more apparent as the SAEO concentration increased [58]. Within the portion of the visible region displayed in Figure 7, the films maintained relatively high transparency despite the observed UV attenuation.

In the UV region (approximately 200–350 nm), SAEO-incorporated PVA films demonstrated markedly lower transmittance compared with neat PVA. This behavior can be attributed to the presence of phenolic compounds, primarily eugenol, which constitutes the major bioactive component of clove essential oil. The aromatic ring and conjugated π-electron system of eugenol exhibit strong UV absorption, thereby enhancing the UV-shielding capability of the polymer matrix. Similar UV-blocking effects induced by phenolic-rich essential oils have been widely reported in biopolymer-based films [59,60].

In the visible region, all samples showed a gradual increase in transmittance; however, SAEO-containing films maintained lower %T values relative to neat PVA. This reduction may be associated with light scattering phenomena arising from the dispersion of essential oil droplets within the PVA matrix, which disrupts optical homogeneity and increases refractive index mismatches. The observed concentration-dependent decrease in transparency suggests that higher SAEO loadings intensify these scattering effects [61,62]. However, slight deviations from ideal proportional attenuation may be attributed to micro-scale dispersion differences or partial surface enrichment of the essential oil, which can influence light scattering efficiency without necessarily reflecting the exact retained oil fraction.

Overall, the results indicate that the incorporation of SAEO effectively tailors the optical properties of PVA films, imparting enhanced UV barrier functionality while maintaining acceptable transparency in the visible region. Such characteristics are particularly advantageous for active and protective food packaging applications, where shielding against UV-induced oxidation and photodegradation is critically important.

### 3.5. Antibacterial Activity

**Figure 8:**
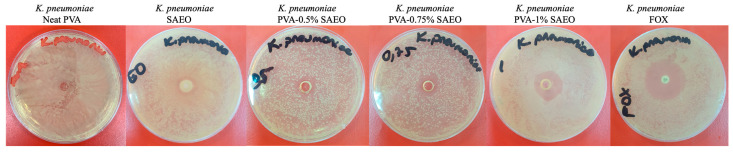
Zones of inhibition of neat PVA, SAEO, antibacterial polymer films (PVA-SAEO 0.5%, PVA-SAEO 0.75%, and PVA-SAEO 1.0%) and FOX as a positive control. Each experimental trial was repeated three times.

This study investigated the antibacterial activity of PVA-SAEO film samples against the bacteria *K. pneumoniae*. The diameter of the resulting inhibition zone has been considered an indicator of antibacterial efficacy. In this method, wider inhibition zones indicate higher antibacterial activity due to more effective diffusion of active ingredients and stronger suppression of bacterial growth. The concentration-dependent increase in inhibition zone diameter shows that the antibacterial efficacy of the films increases as the SAEO content increases, revealing a direct relationship between active ingredient concentration and antimicrobial efficacy. The results are presented in Table 3, and inhibition zones are also shown in Figure 8. According to the results, PVA-SAEO (0.5%), PVA-SAEO (0.75%), and PVA-SAEO (1.0%) films showed antibacterial activity, forming growth inhibition zones (15, 17, and 19 mm, respectively) on *K. pneumoniae*.

Clove oil’s mechanism of action is thought to involve disruption of bacterial cell membranes, biofilm breakdown, and interference with metabolic pathways [63]. A molecular docking study supports these findings by highlighting interactions between key proteins, specifically the *gnd* gene (involved in capsule production) [22] and the *fimH* and *mrkD* genes (which play important roles in biofilm formation) [25], as well as the major components of SAEO, which possesses antibacterial properties. SAEO’s addition to PVA as an additive, as well as the effective antibacterial activity against *K. pneumoniae* that it has demonstrated, indicates that this material is a promising candidate for medical devices.

### 3.6. A Bioinformatics Study on Various Virulence Factors of K. pneumoniae

The potential subcellular location of the 2ZYG protein was predicted to be cytoplasmic, while those predicted for 3U4K and 9AT9 proteins were determined to be extracellular. Accurate prediction of subcellular locations is essential for understanding the functions and interactions of intracellular proteins [64]. Subcellular compartments provide specific physiological and biochemical environments in which proteins can function effectively, facilitating cell growth, energy production, and essential metabolic functions; therefore, understanding where proteins reside within these compartments can provide critical insights into their roles in these biological processes [65]. Extracellular proteins significantly determine bacterial adhesion to various solid surfaces [66]. These secreted proteins accumulate on the cell surface before adsorbing to the contact surface. The protein layer on this surface facilitates bacterial adhesion by anchoring the single cells [67]. The properties of the virulence proteins (PDB ID: 2ZYG, 3U4K, 9AT9) in *K. pneumoniae* are summarized in Table 4.

### 3.7. Molecular Docking

The docking results for the proteins with PDB IDs 2ZYG, 3U4K, and 9AT9 from *K. pneumoniae*, along with the ligands eugenol, eugenylacetate, α-cubebene, guanosine, apioline, β-caryophyllene, and elemicin, were evaluated using root mean square deviation (RMSD) values. The RMSD/upper bound (UB) and RMSD/lower bound (LB) metrics provide insights into the statistical variability of a molecule’s dynamic movements and can help to assess the reliability of molecular dynamics simulation results.

The molecular docking study predicted multiple binding modes. Among these, the binding modes with RMSD/LB and RMSD/UB values of 0 are the most stable and reliable, as summarized in Table 5. A negative binding energy signifies that the free energy of a ligand decreases upon binding to the target molecule, indicating that the binding is thermodynamically favorable. This decrease in free energy during bonding suggests that interactions between the two molecules stabilize a bonding conformation [68].

The strongest binding energies were observed between 2ZYG and α-cubebene (−6.4 kcal/mol), 2ZYG and β-caryophyllene (−6.4 kcal/mol), 3U4K and guanosine (−5.6 kcal/mol), and 9AT9 and guanosine (−5.2 kcal/mol), respectively.

The interaction type, interacting amino acid name, and bond length (Å) obtained as a result of molecular docking between proteins with PDB IDs 2ZYG, 3U4K, and 9AT9 and the ligands eugenol, eugenyl acetate, α-cubebene, guanosine, apioline, β-caryophyllene, and elemicin are summarized in Table 6, which shows that van der Waals interactions are observed between the 2ZYG, 3U4K, and 9AT9 virulence proteins of *K. pneumoniae* and the major compounds of clove. van der Waals forces are decisive in protein–ligand complex formation, and various studies have shown that these interactions are crucial for ligand binding affinity to the protein [69]. This molecular docking study also shows the presence of important bonds, including conventional hydrogen bonds, alkyl, pi-alkyl, pi-sigma, carbon–hydrogen bonds, pi-donor hydrogen bonds, pi-pi stacked, and amide-pi stacked bonds (Table 6).

The presence of conventional hydrogen bonds between the ligand and the receptor indicates a strong, specific interaction between the two molecules. These bonds enhance the stability of the ligand–receptor interaction, enabling specific binding and initiating the biological reaction. Therefore, conventional hydrogen bonds between ligands and receptors play a crucial role in understanding key processes such as cellular signaling and drug interactions in biological systems [70].

Carbon–hydrogen bonds can enhance the binding affinity of a ligand to its target protein. Additionally, ligands containing aromatic rings can form strong interactions with specific regions of the protein surface via π-σ bonds [71]. Types of interactions, such as pi-alkyl bonds, also enhance the ligand’s hydrophobic interactions within the receptor’s binding pocket [72]. Moreover, amide-π stacked bonds, which strengthen ligand–receptor interactions, have been observed in complexes such as 2ZYG/eugenol, 2ZYG/elemicin, and 3U4K/guanosine.

The study also shows that the presence of a π-donor hydrogen bond between 3U4K and apioline contributes to a strong interaction. In other words, π-donor hydrogen bonds increase the stability of binding sites between the ligand and protein [73]. Furthermore, the amino acids involved in these interactions are located within the conserved sequences identified for each protein molecule in Table 4.

**Figure 9:**
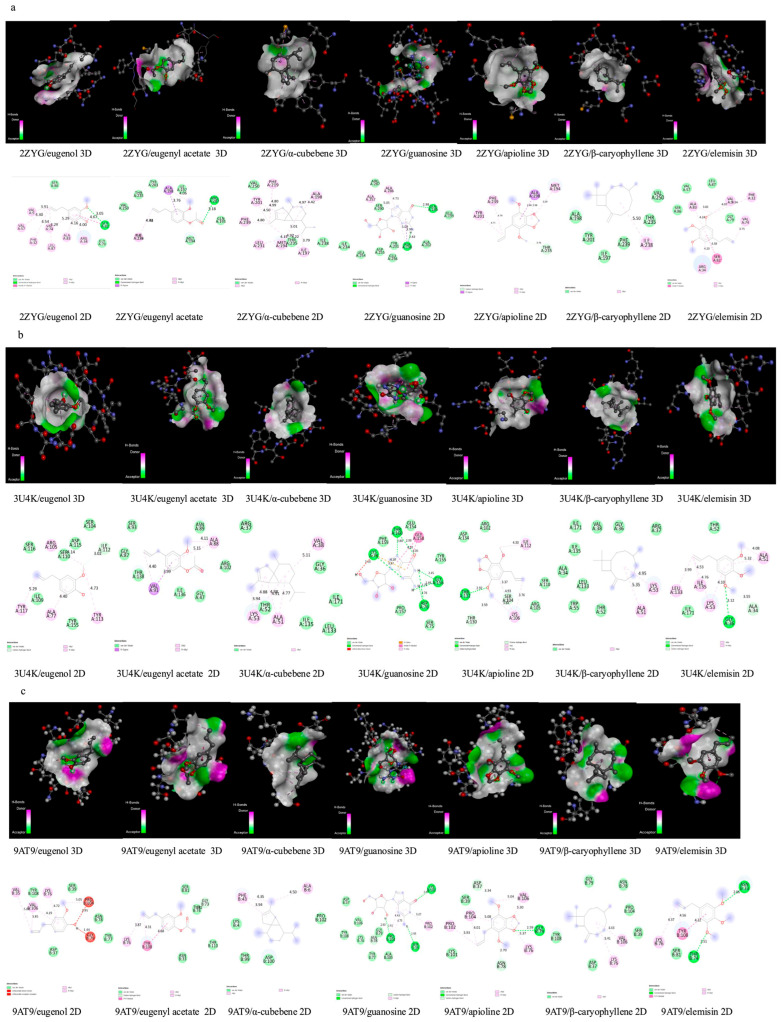
(**a**) Three-dimensional and 2D representations of the interaction between the amino acids in the binding region of the target macromolecule coded 2ZYG and the ligands (eugenol, eugenyl acetate, α-cubebene, guanosine, apioline, β-caryophyllene, and elemicin), (**b**) 3D and 2D representations of the interaction between the amino acids in the binding region of the target macromolecule coded 3U4K and the ligands (eugenol, eugenyl acetate, α-cubebene, guanosine, apioline, β-caryophyllene, and elemicin), (**c**) 3D and 2D representations of the interaction between the amino acids in the binding region of the target macromolecule coded 9AT9 and the ligands (eugenol, eugenyl acetate, α-cubebene, guanosine, apioline, β-caryophyllene, and elemicin).

Figure 9 shows 3D and 2D images of the molecular docking results for interactions between proteins 2ZYG, 3U4K, and 9AT9 and the ligands eugenol, eugenyl acetate, α-cubebene, guanosine, apioline, β-caryophyllene, and elemicin. As seen in Figure 9, the amino acids involved in these interactions are located within the conserved sequences identified for each protein molecule in Table 4.

## 4. Conclusions

In this study, PVA-SAEO films with antibacterial properties were produced. FTIR results of the produced films confirmed that the structural integrity of the PVA matrix was preserved and that the essential oil was physically trapped within the polymer network. DSC results confirmed that SAEO acted as an effective plasticizer in PVA films, significantly altering the glass transition behavior and increasing polymer chain mobility depending on the concentration. DMA-derived TMA results were consistent with the DSC analysis. In conclusion, clove essential oil, classified as safe (GRAS) by the FDA, imparted strong antibacterial properties to PVA without significantly altering its transparency and thermal properties. The results of the molecular docking study support the antibacterial activity against *K. pneumoniae*. This study aims to develop a biocompatible, antibacterial, and renewable material for use in medical device production, where the physicochemical, thermal, and antibacterial findings indicate significant progress toward this goal. However, further biocompatibility, toxicity, and long-term stability studies are needed to verify safe and effective use in medical applications. Therefore, while PVA-SAEO films are potential candidate materials, their clinical potential should be evaluated through further studies.

An interesting finding of the study was that adding clove essential oil effectively altered the optical properties of PVA films, enhancing their UV barrier function while maintaining acceptable transparency in the visible region. These properties suggest that it could be a potential candidate for active and preservative food packaging applications where protection against UV-induced oxidation and photodegradation is critical.

## 5. Study Limitations

In this study, antibacterial evaluation was performed on *Klebsiella pneumoniae*, which was selected as a model Gram-negative bacterium due to its clinical significance as an ESKAPE pathogen and strong biofilm-forming capacity. However, limitations of the study include the fact that only a single microorganism was studied and that contact-based CFU reduction tests, biofilm analyses, and MIC/MBC determinations were not performed.

Comprehensive validation analyses for specific application areas such as medical devices or food packaging (biocompatibility, toxicity, long-term safety, mechanical and barrier properties, and migration/release tests) are beyond the scope of this study. The current research focused on developing the relevant material and elucidating its basic structural, thermal, and antibacterial properties, and detailed validation studies for the application are planned as part of further research.

## Figures and Tables

**Table 2:** The elastic modulus values of samples calculated with the creep-converted stress–strain curves.

Sample	Elastic Modulus (MPa)
PVA	1849 ± 245
PVA-0.50SAEO	1584 ± 166
PVA-0.75SAEO	1602 ± 97
PVA-1.00SAEO	1581 ± 205

**Table 3:** Antibacterial activity of the films against *K. pneumoniae*.

Treatments	Inhibition Zone (mm) on *K. pneumoniae*
PVA-SAEO (0.5%)	15
PVA-SAEO (0.75%)	17
PVA-SAEO (1%)	19
SAEO (30 µL/well)	14
FOX ^a^	35

^a^ FOX: cefoxitin (30 µg/mL).

**Table 4:** The protein features of virulence factors in *K. pneumoniae*.

Bacteria	PDB ID	Protein Names	Gene Name	CD	Proteins (aa) Lengths from NCBI GenBank	SL
*K. pneumoniae*	2ZYG	gluconate-6-phosphate dehydrogenase	*gnd*	2-466	468	C
	3U4K	mrkD	*mrkD*	1-321	321	EC
	9AT9	FimH	*fimH*	27-302	302	EC

CD: conserved domain, EC: extracellular, C: cytoplasmic, SL: subcellular location, NCBI: The National Center for Biotechnology Information.

**Table 5:** Energy and RMSD values obtained during docking analysis of ligand molecules and target proteins.

Complex	BE	RMSD/UB	RMSD/LB
2ZYG/eugenol	−5.4	0.00	0.00
2ZYG/eugenyl acetate	−5.3	0.00	0.00
2ZYG/α-cubebene	−6.4	0.00	0.00
2ZYG/guanosine	−6.0	0.00	0.00
2ZYG/apioline	−5.0	0.00	0.00
2ZYG/β-caryophyllene	−6.4	0.00	0.00
2ZYG/elemicin	−4.9	0.00	0.00
3U4K/eugenol	−4.7	0.00	0.00
3U4K/eugenyl acetate	−4.8	0.00	0.00
3U4K/α-cubebene	−5.3	0.00	0.00
3U4K/guanosine	−5.6	0.00	0.00
3U4K/apioline	−4.4	0.00	0.00
3U4K/β-caryophyllene	−5.0	0.00	0.00
3U4K/elemicin	−4.4	0.00	0.00
9AT9/eugenol	−4.4	0.00	0.00
9AT9/eugenyl acetate	−4.6	0.00	0.00
9AT9/α-cubebene	−5.0	0.00	0.00
9AT9/guanosine	−5.2	0.00	0.00
9AT9/apioline	−4.4	0.00	0.00
9AT9/β-caryophyllene	−4.9	0.00	0.00
9AT9/elemicin	−4.1	0.00	0.00

**Table 6:** The molecular docking parameters between 2ZYG, 3U4K, and 9AT9 and the ligands.

Protein–Ligand	Interaction Type	Interacting Amino Acid	Bond Length (Å)
2ZYG/eugenol	van der Waals	GLY79, SER86	-
	Conventional Hydrogen Bonds	SER82	3.05
	Amide-Pi Stacked	SER82:C,O;ALA83:N	4.67
	Alkyl	VAL9, VAL57, LEU87	3.91, 4.30, 4.29
	Pi-Alkyl	LEU32, ARG34, VAL74, ALA83	4.54, 4.00, 5.29, 4.16
2ZYG/eugenyl acetate	van der Waals	MET194, GLN195, ILE197, TYR201, THR235, VAL250	-
	Conventional Hydrogen Bonds	ARG287	3.18
	Pi-Sigma	ALA198	3.76
	Alkyl	ALA198, ILE238	4.06, 4.71
	Pi-Alkyl	PHE239	4.44
2ZYG/α-cubebene	van der Waals	ILE234, ILE238, THR235, VAL230	-
	Alkyl	MET194, ALA198, ALA198, ILE197, MET194, ILE197, LEU231	5.01, 4.97, 4.42, 4.96, 4.22, 3.79, 4.15
	Pi-Alkyl	TYR201, TYR201, PHE219, PHE239	4.99, 4.50, 4.80, 4.80
2ZYG/guanosine	van der Waals	GLU199, LEU254, ASP255, GLU256, TYR201, ALA202, ARG287, SER290	-
	Conventional Hydrogen Bonds	GLN195, ALA198	2.98, 2.82
	Pi-Sigma	ALA198	3.99
	Pi-Alkyl	ALA198, ALA257, ALA286	5.09, 5.05, 4.71
2ZYG/apioline	van der Waals	ILE238, ILE238, VAL250	-
	Carbon–Hydrogen Bonds	THR235	3.76
	Pi-Sigma	ALA198	3.48
	Alkyl	ALA198, MET194	3.98, 5.09
	Pi-Alkyl	TYR201, PHE239	4.71, 4.79
2ZYG/β-caryophyllene	van der Waals	ILE197, ALA198, TYR201, THR235, PHE239, VAL250	-
	Alkyl	ILE238	5.50
2ZYG/elemicin	van der Waals	VAL57, GLY79, SER86, LEU87	-
	Amide-Pi Stacked	SER82:C,O;ALA83:N	4.38
	Alkyl	ALA83, VAL74, VAL9	3.82, 3.75, 4.02
	Pi-Alkyl	PHE32, ARG34, ALA83	4.94, 4.20, 4.24
3U4K/eugenol	van der Waals	SER104, ILE109, SER110, ASP115, SER116, TYR155	-
	Carbon–Hydrogen Bonds	ILE112	3.02
	Alkyl	ARG105	4.14
	Pi-Alkyl	TYR113, TYR117, ALA77	4.73, 5.29, 4.40
3U4K/eugenyl acetate	van der Waals	GLY47, GLY87, ASN89, SER93, ARG102, ILE136, THR138	-
	Pi-Sigma	VAL91	3.99
	Alkyl	ALA88, VAL91	4.11, 4.40
	Pi-Alkyl	ALA88	5.15
3U4K/α-cubebene	van der Waals	GLY36, ARG37, THR52, LEU133, ILE135, ILE171	-
	Alkyl	VAL38, ALA51, ALA51, LYS53, LYS53, LYS53	5.11, 4.91, 4.77, 4.88, 4.98, 3.94
3U4K/guanosine	van der Waals	SER75, GLU154, TYR155, PRO157, PHE159	-
	Conventional Hydrogen Bonds	LYS78, THR160, THR160, ASP76, GLY156, ASP76, GLY156	3.37, 2.99, 2.80, 2.79, 2.45, 2.76, 2.49
	Pi-Cation	LYS78	4.18
	Amide-Pi Stacked	GLY158:C,O;PHE159:N GLY158:C,O;PHE159:N	4.20, 4.05
	Pi-Alkyl	LYS78	4.96
3U4K/apioline	van der Waals	ARG102, ARG105, SER110, ASP134	-
	Conventional Hydrogen Bonds	THR132	2.92
	Carbon–Hydrogen Bonds	THR130	3.59
	Pi-Donor Hydrogen Bonds	SER104	3.37
	Alkyl	LYS106, ILE112, LYS106	3.76, 4.30, 3.84
	Pi-Alkyl	LYS106	4.93
3U4K/β-caryophyllene	van der Waals	ALA34, GLY36, ARG37, VAL38, THR52, TRP55, LEU133, ILE135, ILE171	-
	Alkyl	ALA51, LYS53	5.35, 4.95
3U4K/elemicin	van der Waals	THR52, ILE171	-
	Conventional Hydrogen Bonds	GLY36	3.12
	Carbon–Hydrogen Bonds	ALA34	3.55
	Alkyl	ALA51, LYS53, LEU133, ILE135	4.08, 4.76, 3.99, 4.53
	Pi-Alkyl	ALA51, LYS53	5.32, 4.10
9AT9/eugenol	van der Waals	ASP37, SER39, TYR77, ASN78, TYR108	-
	Alkyl	VAL35, VAL106, PRO104	5.01, 3.85, 5.05
	Pi-Alkyl	LYS76, VAL106	4.72, 4.19
9AT9/eugenyl acetate	van der Waals	ASN33, THR74, SER81	-
	Carbon–Hydrogen Bonds	GLY73	2.82
	Pi-Pi Stacked	TYR108	4.68
	Alkyl	LYS76	3.87
	Pi-Alkyl	TYR108	4.31
9AT9/α-cubebene	van der Waals	LYS4, THR99, ASP100, PRO102	-
	Alkyl	ALA6	4.50
	Pi-Alkyl	PHE43, PHE43	3.94, 4.35
9AT9/guanosine	van der Waals	ASP37, LYS76, TYR77, GLY79, ALA106, VAL106, TYR108	-
	Conventional Hydrogen Bonds	LYS101, SER39, PRO104	2.57, 2.55, 2.51
	Carbon–Hydrogen Bonds	ASN78	2.93
	Pi-Alkyl	PRO104, PRO102, PRO104	4.43, 5.07, 4.72
9AT9/apioline	van der Waals	SER39, LYS101	-
	Conventional Hydrogen Bonds	GLY79	2.59
	Carbon–Hydrogen Bonds	ASN78, ASP37	2.70, 3.34
	Alkyl	LYS76, VAL106, PRO102, PRO104, VAL106	5.37, 5.30, 3.93, 4.01, 5.04
	Pi-Alkyl	PRO104	5.08
9AT9/β-caryophyllene	van der Waals	ASP37, SER39, ASN78, GLY79, PRO104, TYR108	-
	Alkyl	LYS76, VAL106	5.41, 4.66
9AT9/elemicin	van der Waals	SER81	-
	Conventional Hydrogen Bonds	ASN33, THR74	2.96, 2.51
	Pi-Pi Stacked	TYR108	4.22
	Alkyl	LYS76	4.37
	Pi-Alkyl	TYR108	4.56

## Data Availability

The contributions presented in this study are included in this article. For further inquiries, please contact the corresponding author.

## References

[1] Mustafa G., Arif R. (2025). Introductory Chapter: Antimicrobial Resistance–Historical Perspectives and Future Directions. Antimicrobial Resistance-New Insights. Antimicrobial Resistance-New Insights.

[2] Ahmed S.K., Hussein S., Qurbani K., Ibrahim R.H., Fareeq A., Mahmood K.A., Mohamed M.G. (2024). Antimicrobial Resistance: Impacts, Challenges, and Future Prospects. J. Med. Surg. Public Health.

[3] Oliveira M., Antunes W., Mota S., Madureira-Carvalho Á., Dinis-Oliveira R.J., Dias da Silva D. (2024). An overview of the recent advances in antimicrobial resistance. Microorganisms.

[4] Aijaz M., Ahmad M., Ansari M.A., Ahmad S. (2023). Antimicrobial Resistance in a Globalized World: Current Challenges and Future Perspectives. Int. J. Pharm. Drug Des..

[5] Thong C.C., Teo D.C.L., Ng C.K. (2016). Application of polyvinyl alcohol (PVA) in cement-based composite materials: A review of its engineering properties and microstructure behavior. Constr. Build. Mater..

[6] Saeed A., Guizani I., Hanash F.E., Asnag G.M., Al-Harthi A.M., Alwafi R., Assran A.S. (2024). Enhancing Optical, Structural, Thermal, Electrical Properties, and Antibacterial Activity in Chitosan/Polyvinyl Alcohol Blend with ZnO Nanorods: Polymer Nanocomposites for Optoelectronics and Food/Medical Packaging Applications. Polym. Bull..

[7] Ağçeli G.K. (2024). Polymer and its nanocomposites as an antimicrobial coating for medical devices and implants. Next-Generation Antimicrobial Nanocoatings for Medical Devices and Implants.

[8] Jiang J., Zhang W., Yi X., Lei Q., Liao Y., Tan Y., Yu W. (2024). Recent progress in properties and application of antibacterial food packaging materials based on polyvinyl alcohol. e-Polymers.

[9] Amalraj A., Haponiuk J.T., Thomas S., Gopi S. (2020). Preparation, characterization and antimicrobial activity of polyvinyl alcohol/gum arabic/chitosan composite films incorporated with black pepper essential oil and ginger essential oil. Int. J. Biol. Macromol..

[10] Altaf F., Niazi M.B.K., Jahan Z., Ahmad T., Akram M.A., Safdar A., Butt M.S., Noor T., Sher F. (2021). Synthesis and Characterization of PVA/Starch Hydrogel Membranes Incorporating Essential Oils Aimed to be Used in Wound Dressing Applications. J. Polym. Environ..

[11] Ünlü N., Özgen A., Aksu Canbay C. (2024). Production and characterization of polyvinyl alcohol films containing essential oil. J. Biomater. Sci. Polym. Ed..

[12] Yeşilyurt A. (2025). Development and Characterization of PVA/KGM-Based Bioactive Films Incorporating Natural Extracts and Thyme Oil. Polymers.

[13] Kumar V., Mishra D., Joshi M.C., Mishra P., Tanwar M. (2021). Herbs and Spices—New Processing Technologies. *Syzygium aromaticum*: Medicinal Properties and Phytochemical Screening. Herbs and Spices-New Processing Technologies.

[14] Kumar-Pandey V., Shams R., Singh R., Dar A.H., Pandiselvam R., Rusu A.V., Trif M. (2022). A Comprehensive Review on Clove (*Caryophyllus aromaticus* L.) Essential Oil and Its Significance in the Formulation of Edible Coatings for Potential Food Applications. Front. Nutr..

[15] Chen X., Ren L., Li M., Qian J., Fan J., Du B. (2017). Effects of clove essential oil and eugenol on quality and browning control of fresh-cut lettuce. Food Chem..

[16] Abbas R., Chakkour M., Zein El Dine H., Obaseki E.F., Obeid S.T., Jezzini A., Ghssein G., Ezzeddine Z. (2024). General Overview of *Klebsiella pneumonia*: Epidemiology and the Role of Siderophores in Its Pathogenicity. Biology.

[17] Wei Y., Shi D., Chen T., Zhou S., Yang Z., Li H., Jin M. (2024). Hypervirulent *Klebsiella pneumoniae* with a hypermucoviscosity phenotype challenges strategies of water disinfection for its capsular polysaccharides. Water Res..

[18] Sati H., Carrara E., Savoldi A., Hansen P., Garlasco J., Campagnaro E., Boccia S., Castillo-Polo J.A., Magrini E., Garcia-Vello P. (2025). The WHO Bacterial Priority Pathogens List 2024: A Prioritisation Study to Guide Research, Development, and Public Health Strategies against Antimicrobial Resistance. Lancet Infect. Dis..

[19] Xu L., Li J., Wu W., Wu X., Ren J. (2024). *Klebsiella pneumoniae* Capsular Polysaccharide: Mechanism in Regulation of Synthesis, Virulence, and Pathogenicity. Virulence.

[20] Shu H.Y., Fung C.P., Liu Y.M. (2009). Genetic diversity of capsular polysaccharide biosynthesis in *Klebsiella pneumoniae* clinical isolates. Microbiology.

[21] Panjaitan N.S., Horng Y.T., Chien C.C., Yang H.C., You R.I., Soo P.C. (2021). The PTS Components in *Klebsiella pneumoniae* Affect Bacterial Capsular Polysaccharide Production and Macrophage Phagocytosis Resistance. Microorganisms.

[22] Mendes G., Santos M.L., Ramalho J.F., Duarte A., Caneiras C. (2023). Virulence factors in carbapenem-resistant hypervirulent *Klebsiella pneumoniae*. Front. Microbiol..

[23] Nelson K., Selander R.K. (1994). Intergeneric transfer and recombination of the6-phosphogluconate dehydrogenase gene (*gnd*) in enteric bacteria. Proc. Natl. Acad. Sci. USA.

[24] Davoudabadi S., Goudarzi M., Hashemi A. (2023). Detection of virulence factors and antibiotic resistance among *Klebsiella pneumoniae* isolates from Iran. BioMed Res. Int..

[25] Riwu K.H.P., Effendi M.H., Rantam F.A., Khairullah A.R., Widodo A. (2022). A review: Virulence factors of *Klebsiella pneumonia* as emerging infection on the food chain. Vet. World.

[26] Paczosa M.K., Mecsas J. (2016). *Klebsiella pneumoniae*: Going on the Offense with a Strong Defense. Microbiol. Mol. Biol. Rev..

[27] Bryers J.D. (2008). Medical biofilms. Biotechnol. Bioeng..

[28] Ortega-Portas C., Esteban J. (2025). New strategies for the management of biofilms formed by Gram-negative bacteria. Expert Opin. Pharmacother..

[29] Esakkimuthu S., Ganga V.S., Arumugam A., Subramaniam S. (2025). Targeting Extracellular Polymeric Substances and Multidrug-Resistant Bacteria Biofilms Using Zinc Oxide–Enhanced Chitosan/Polyvinyl Alcohol Nanofibers Deposited on Bacterial Cellulose. APMIS.

[30] Canbay C.A., Ünlü N. (2021). Production and characterization of shape memory polymeric nanocomposite materials. J. Mol. Struct..

[31] Pugar D., Haramina T., Leskovac M., Curkovic L. (2024). Preparation and Characterization of Poly(vinyl-alcohol)/Chitosan Polymer Blend Films Chemically Crosslinked with Glutaraldehyde: Mechanical and Thermal Investigations. Molecules.

[32] Shan Z., Huang J., Huang Y., Zhou Y., Li Y. (2024). Glutaraldehyde crosslinked ternary carboxymethylcellulose/polyvinyl alcohol/polyethyleneimine film with enhanced mechanical properties, water resistance, antibacterial activity, and UV-shielding ability without any UV absorbents. Int. J. Biol. Macromol..

[33] Durmus Z., Köferstein R., Özgen A., Lindenberg T., Maijenburg A.W., Durmus A. (2025). Novel and Highly Efficient Antibacterial PLA Composites Prepared with *Liquidambar Orientalis* Oil and Ag@g-C3N4 Nanocomposite. J. Polym. Environ..

[34] Pınar O., Koc F.E., Alanalp M.B., Sivri N., Ezdesir A., Durmus A. (2024). Utilization of Silybum marianum extract as a high-performance natural antioxidant for polyethylene. J. Mater. Sci..

[35] CLSI (2012). Performance Standards for Antimicrobial Susceptibility Testing: Twenty-fifth Informational Supplement—CLSI Document M100-S25.

[36] Sayed A., Gehan S., Manar A., Ghada A.M. (2023). Alkali-cellulose/Polyvinyl alcohol biofilms fabricated with essential clove oil as a novel scented antimicrobial packaging material. Carbohydr. Polym. Technol. Appl..

[37] Sharma H., Pathak M. (2024). Development of PCL/TiO2 composite as an efficient antibacterial, anticancer drug and biocompatible properties. Results Chem..

[38] Ayodele P.F., Bamigbade A., Bamigbade O.O., Adeniyi I.A., Tachin E.S., Seweje A.J., Farohunbi S.T. (2023). Illustrated Procedure to Perform Molecular Docking Using PyRx and Biovia Discovery Studio Visualizer: A Case Study of 10kt With Atropine. Prog. Drug Discov. Biomed. Sci..

[39] Coates J. (2006). Interpretation of Infrared Spectra, A Practical Approach. Encyclopedia of Analytical Chemistry: Applications, Theory and Instrumentation.

[40] Hassan C.M., Peppas N.A. (2000). Structure and Applications of Poly(vinyl alcohol) Hydrogels Produced by Conventional Crosslinking or by Freezing/Thawing Methods. Biopolymers PVA Hydrogels, Anionic Polymerisation Nanocomposites.

[41] Sánchez-González L., Chiralt A., González-Martínez C., Cháfer M. (2011). Effect of essential oils on properties of film forming emulsions and films based on hydroxypropylmethylcellulose and chitosan. J. Food Eng..

[42] Marchese A., Barbieri R., Coppo E., Orhan I.E., Daglia M., Nabavi S.F., Izadi M., Abdollahi M., Nabavi S.M., Ajami M. (2017). Antimicrobial activity of eugenol and essential oils containing eugenol: A mechanistic viewpoint. Crit. Rev. Microbiol..

[43] Maria T.M., Carvalho R.A., Sobral P.J., Habitantea A.M., Solorza-Feriab J. (2008). The effect of the degree of hydrolysis of the PVA and the plasticizer concentration on the color, opacity, and thermal and mechanical properties of films based on PVA and gelatin blends. J. Food Eng..

[44] Sarti B., Scandola M. (1995). Viscoelastic and thermal properties of collagen/poly(vinyl alcohol) blends. Biomaterials.

[45] Tsioptsias C., Fardis D., Ntampou X., Tsivintzelis I., Panayiotou C. (2023). Thermal Behavior of Poly (vinyl alcohol) in the Form of Physically Crosslinked Film. Polymers.

[46] Fan Y., Xiao X., Zou Y., Ren J., Zhang Q., Yang Q., Liu F. (2025). Polyvinyl alcohol-based sustainable packaging films incorporating glycerol monostearate: Fabrication, characterization, and intermolecular interactions analysis. Food Wellness.

[47] Panova T.V., Efimova A.A., Berkovich A.K., Efimov A.V. (2020). Plasticity control of poly(vinyl alcohol)–graphene oxide nanocomposites. RSC Adv..

[48] Sallam A.S., Sallam M.S., Abdin M., Salem M.E., Xie Z., Fan X., Zeng X. (2025). Development of eco-friendly PVA/Sodium alginate/carboxymethyl cellulose films enhanced with γ-oryzanol and clove oil as active packaging materials for oily foods preservation. Future Foods.

[49] Lim W.S., Kim M.H., Park H.J., Lee M.H. (2024). Characterization of Polyvinyl Alcohol (PVA)/Polyacrylic Acid (PAA) Composite Film-Forming Solutions and Resulting Films as Affected by Beeswax Content. Polymers.

[50] Finch C.A. (1974). Polyvinyl Alcohol, Properties and Applications. J. Polym. Sci. Polym. Lett. Ed..

[51] Tadmor Z., Gogos C.G. (2006). Principles of Polymer Processing.

[52] Sothornvit R., Krochta J.M. (2001). Plasticizer effect on mechanical properties of β-lactoglobulin films. J. Food Eng..

[53] Noori S.M.A., Hossaeini Marashi S.M. (2023). Chitosan-based coatings and films incorporated with essential oils: Applications in food models. J. Food Meas. Charact..

[54] Vieira M.G.A., da Silva M.A., Dos Santos L.O., Beppu M.M. (2011). Natural-based plasticizers and biopolymer films: A review. Eur. Polym. J..

[55] Menard K.P., Menard N. (2020). Dynamic Mechanical Analysis.

[56] Zhou Y., Wu X., Chen J., He J. (2021). Effects of Cinnamon Essential Oil on the Physical, Mechanical, Structural and Thermal Properties of Cassava Starch-Based Edible Films. Int. J. Biol. Macromol..

[57] Aydın A.A., Ilberg V. (2016). Effect of different polyol-based plasticizers on thermal properties of polyvinyl alcohol:starch blends. Carbohydr. Polym..

[58] Sánchez-González L., Cháfer M., Chiralt A., González-Martínez C. (2010). Physical Properties of Edible Chitosan Films Containing Bergamot Essential Oil and Their Inhibitory Action on Penicillium Italicum. Carbohydr. Polym..

[59] Atarés L., Chiralt A. (2016). Essential Oils as Additives in Biodegradable Films and Coatings for Active Food Packaging. Trends Food Sci. Technol..

[60] Liñán-Atero R., Aghababaei F., García S.R., Hasiri Z., Ziogkas D., Moreno A., Hadidi M. (2024). Clove Essential Oil: Chemical Profile, Biological Activities, Encapsulation Strategies, and Food Applications. Antioxidants.

[61] Llana-Ruiz-Cabello M., Pichardo S., Bãnos A., Núñez C., Bermúdez J.M., Guillamón E., Aucejo S., Cameán A.M. (2015). Characterisation and evaluation of PLA films containing an extract of Allium spp. to be used in the packaging of ready-to-eat salads under controlled atmospheres. LWT Food Sci. Technol..

[62] Ghanbarzadeh B., Almasi H. (2011). Physical properties of edible emulsified films based on carboxymethyl cellulose and oleic acid. Int. J. Biol. Macromol..

[63] Bhattacharjee A., Jamal T.B., Ahammad I., Chowdhury Z.M., Rahman A., Dewan G. (2023). Shotgun metagenomics unravels higher antibiotic resistome profile in Bangladeshi gut microbiome. bioRxiv.

[64] Moreno J., Nielsen H., Winther O., Teufel F. (2024). Predicting the subcellular location of prokaryotic proteins with DeepLocPro. Bioinformatics.

[65] Zeng G.H., Zhu X.Z., Yang H.R., Liang Y.J., Zhai Y.J., Xu Y.Y. (2025). Knowledge-enhanced protein subcellular localization prediction from 3D fluorescence microscope images. Bioinformatics.

[66] Fong J.N.C., Yildiz F.H. (2015). Biofilm Matrix Proteins. Microb. Biofilms.

[67] Vandana, Das S. (2022). Genetic regulation, biosynthesis and applications of extracellular polysaccharides of the biofilm matrix of bacteria. Carbohydr. Polym..

[68] Kawsar S.M., Hossain M.A., Saha S., Abdallah E.M., Bhat A.R., Ahmed S., Jamalis J., Ozeki Y. (2024). Nucleoside-based drug target with general antimicrobial screening and specific computational studies against SARS-CoV-2 main protease. ChemistrySelect.

[69] Shahraki S., Razmara Z., Delarami H.S., Poorsargol M. (2023). Probing the combination of erlotinib hydrochloride, an anticancer drug, and human serum albumin: Spectroscopic, molecular docking, and molecular dynamic analyses. Luminescence.

[70] Abelian A., Wallach J., Gaye B., Adejare A., Adejare A. (2021). Chapter 6—Pharmaceutical chemistry. Remington.

[71] Singh A., Gogoi H.P., Barman P. (2022). Comparative study of palladium (II) complexes bearing tridentate ONS and NNS Schiff base ligands: Synthesis, characterization, DFT calculation, DNA binding, bioactivities, catalytic activity, and molecular docking. Polyhedron.

[72] Arthur D.E., Uzairu A. (2019). Molecular docking studies on the interaction of NCI anticancer analogues with human Phosphatidylinositol 4,5-bisphosphate 3-kinase catalytic subunit. J. King Saud Univ. Sci..

[73] Sagaama A., Issaoui N. (2020). Design, molecular docking analysis of an anti-inflammatory drug, computational analysis and intermolecular interactions energy studies of 1-benzothiophene-2-carboxylic acid. Comput. Biol. Chem..

